# Surgical spectral imaging

**DOI:** 10.1016/j.media.2020.101699

**Published:** 2020-07

**Authors:** Neil T. Clancy, Geoffrey Jones, Lena Maier-Hein, Daniel S. Elson, Danail Stoyanov

**Affiliations:** a Wellcome/EPSRC Centre for Interventional and Surgical Sciences (WEISS)*,* University College London*,* United Kingdom; b Centre for Medical Image Computing (CMIC), Department of Medical Physics and Biomedical Engineering, University College London, United Kingdom; c Centre for Medical Image Computing (CMIC), Department of Computer Science, University College London, United Kingdom; d German Cancer Research Centre (DKFZ), Heidelberg, Germany; e Hamlyn Centre for Robotic Surgery, Institute of Global Health Innovation, Imperial College London, United Kingdom; f Department of Surgery and Cancer, Imperial College London, United Kingdom

**Keywords:** Multispectral imaging, Hyperspectral imaging, Minimally-invasive surgery, Computational imaging, AI, Artificial intelligence, AOTF, Acousto-optic tuneable filter, CNN, Convolutional neural network, CT, Computed tomography, DMD, Digital micromirror device, DPF, Differential pathlength factor, EMCCD, Electron-multiplying charge-coupled device, FIGS, Fluorescence image-guided surgery, FWHM, Full-width at half-maximum, GI, Gastrointestinal, HSI, Hyperspectral imaging, INN, Invertible neural network, LCTF, Liquid crystal tuneable filter, LED, Light emitting diode, LOOCV, Leave-one-out cross validation, MIS, Minimally-invasive surgery, MRI, Magnetic resonance imaging, MSI, Multispectral imaging, NBI, Narrowband imaging, NIR, Near infrared, OEM, Original equipment manufacturer, RGB, Red, green, blue, sCMOS, Scientific complementary metal-oxide-semiconductor, SFDI, Spatial frequency domain imaging, SNR, Signal-to-noise ratio, SSI, Surgical spectral imaging, SVM, Support vector machine, VOF, Variable optical filter

## Abstract

Recent technological developments have resulted in the availability of miniaturised spectral imaging sensors capable of operating in the multi- (MSI) and hyperspectral imaging (HSI) regimes. Simultaneous advances in image-processing techniques and artificial intelligence (AI), especially in machine learning and deep learning, have made these data-rich modalities highly attractive as a means of extracting biological information non-destructively. Surgery in particular is poised to benefit from this, as spectrally-resolved tissue optical properties can offer enhanced contrast as well as diagnostic and guidance information during interventions. This is particularly relevant for procedures where inherent contrast is low under standard white light visualisation. This review summarises recent work in surgical spectral imaging (SSI) techniques, taken from Pubmed, Google Scholar and arXiv searches spanning the period 2013–2019. New hardware, optimised for use in both open and minimally-invasive surgery (MIS), is described, and recent commercial activity is summarised. Computational approaches to extract spectral information from conventional colour images are reviewed, as tip-mounted cameras become more commonplace in MIS. Model-based and machine learning methods of data analysis are discussed in addition to simulation, phantom and clinical validation experiments. A wide variety of surgical pilot studies are reported but it is apparent that further work is needed to quantify the clinical value of MSI/HSI. The current trend toward data-driven analysis emphasises the importance of widely-available, standardised spectral imaging datasets, which will aid understanding of variability across organs and patients, and drive clinical translation.

## Highlights

• Wider sensor availability and miniaturisation are pushing speed/resolution limits.
• Small surgical datasets exist in many specialities but no standard format.
• Data-driven analysis avoids modelling, improves speed, addresses uncertainty.
• RGB-based functional imaging could exploit existing cameras, chip-on-tip devices.
• Clinical validation with standardised devices and data needed for translation.

## Graphical abstract

**Figure:**
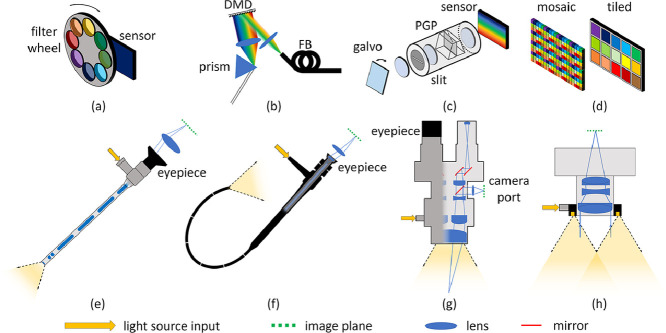

## 1 Introduction

Advances in interventional and surgical techniques have been driven by technological developments in instrumentation and imaging that have enhanced the surgeon's ability to diagnose and treat patients with greater precision. Continuous advances in illumination, detection and display technology are beginning to address limitations and enhance the information available to the clinician beyond that naturally observable by the human eye or under conventional white light visualisation. Spectrally-resolved measurements of reflected light offer a particular opportunity to explore and exploit inherent contrast between different tissues and pathologies during both open and minimally-invasive surgery (MIS). This review summarises the clinical context for this technology, recent hardware and software advances, and discusses how it may be translated into practice.

### 1.1 Biophotonics and surgery

**Fig. 1:**
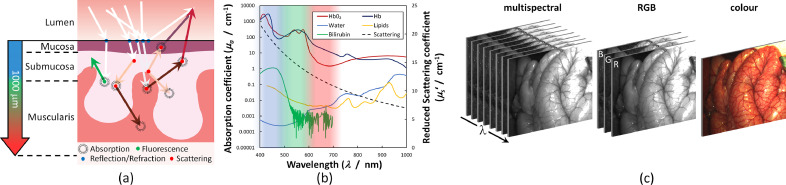
(a) Light propagation in a multi-layered tissue such as the bowel. Some light is regularly reflected, with the rest penetrating the surface where it may be scattered and absorbed (indicated by the shift in colour of the arrows). Autofluorescence emission may also occur. After sufficient scattering a fraction may re-emerge from the tissue and be detected. Red wavelengths penetrate the tissue significantly deeper, due to lower absorption by haemoglobin, than blue or green. (b) Optical properties of major absorbers in surgical imaging applications (absorption data taken from compilation by Prahl (2018)). Haemoglobin (molar concentration 2.33 mM) is the overwhelming chromophore, while the water contribution (55 M) is negligible in the visible range. Bilirubin (20.5 μM) has a peak in the blue but normal physiological concentrations are low. The main lipid (molar concentration unknown, see van Veen et al. (2004) for details) absorption peaks are found at blue and NIR wavelengths. The scattering curve shown, with values indicated on the secondary axis, is constructed using Eq. (2) and typical values for bowel tissue from Jacques (2013). Shaded areas represent typical RGB filter coverage in the visible range. (c) Eight-band MSI datacube of a segment of porcine bowel tissue. The same information is collected by colour cameras in three 100 nm-wide red, green and blue bands (RGB) which, when combined, produce a colour image.

When light penetrates the surface of tissue it may be absorbed, scattered or transmitted. Additionally, after absorption, some molecules may reradiate fluorescent light of longer wavelength (Fig. 1(a)). Therefore, when light is reflected from tissue it carries the fingerprint of the molecular make-up and constituents of that tissue. Other weaker interactions, such as inelastic scattering (Lin et al., 2018a), and non-linear processes may offer additional contrast (Palero et al., 2011), but are not considered here. Fig. 1(b) shows the chief attenuators of light in surgically-exposed tissue, i.e., under the skin. Absorption by oxygenated and deoxygenated haemoglobin (HbO_2_ and Hb, respectively) is dominant, while fat (lipids) has a small peak in the visible range. Water has a much weaker effect, that increases beyond NIR wavelengths. Yellow pigments like bilirubin have an appreciable peak in the blue-green region of the spectrum (Jacques, 2013) but, outside of areas with high concentrations of bile (e.g., the gall bladder (Wirkert et al., 2017) or in cases of bilirubinaemia (Halder et al., 2019)), the volume fraction is low. Elastic scattering occurs due to refractive index discontinuities at a range of length scales from nanometres (cell membrane) to hundreds of microns (fat cells) (Tuchin, 2015a). The strength of these interactions varies with wavelength, meaning that spectrally-resolved measurements may be used to unmix and quantify the individual contributors. The field of biophotonics seeks to accomplish this disentanglement through controlled illumination and detection hardware in tandem with mathematical modelling, simulation and data-driven analysis of the light-tissue interaction.

### 1.2 Optical imaging in surgery

**Fig. 2:**
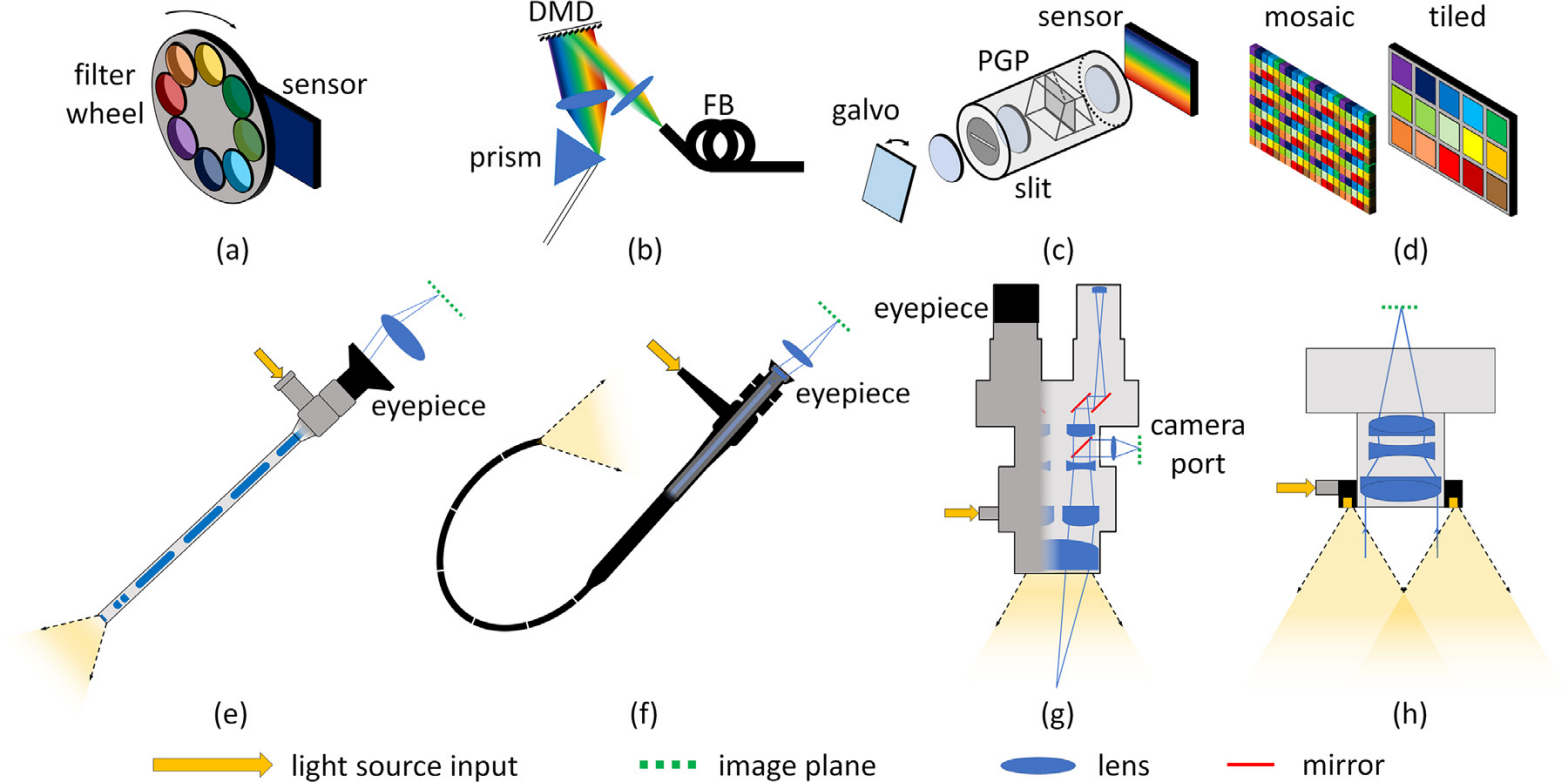
Representative selection of spectral detection mechanisms: (a) Spectral scanning using a filter wheel (or, alternatively, an LCTF/AOTF, Section 2.1) in front of a camera sensor; (b) Spectral scanning using tuneable light source comprised of broadband light dispersed onto a DMD (alternatively, a filter wheel, monochromator or multi-LED source, Section 2.1) and coupled into a fibre bundle (FB); (c) Spatial scanning with a hyperspectral line sensor. The galvo mirror scans the image over the entrance slit to a prism-grating-prism (PGP) spectrograph (or other imaging spectrograph or variable filter, Section 2.2); (d) Snapshot sensors with mosaic and tiled filter arrangements attached directly onto the sensor (for other field-splitting arrangements see Section 2.3). Commonly-used clinical imaging devices, excluding chip-on-tip instruments, are shown, with cross-sections illustrating imaging mechanisms: (e) laparoscope with rod lens image relay and optical fibres running inside the shaft to carry illumination light; (f) fibrescope with flexible fibre image guide and fibre optic illumination; (g) operating microscope with co-axial illumination; (h) externally-mounted imager for open surgery using camera lens and ring light illumination. The lenses at the eyepiece/camera port must be chosen to form a suitably-magnified image on the sensor, if employing SSI types (a, d), or to produce parallel rays, if using an additional scanning mechanism as in (c).

Colour digital cameras are the primary sensing tools used to guide surgeons during MIS (Mirota et al., 2011). These mimic the sensitivity of the human eye by detecting visible wavelengths of light in the red, green and blue (RGB) spectral regions. These are mounted on the proximal end of rigid endoscopes (Fig. 2(e)), which relay images of the tissue *via* a system of Hopkins rod lenses, while illumination is provided by a high-brightness white light source (Cockett and Cockett, 1998). Flexible endoscopes (and, recently, some rigid endoscopes) have distal tip-mounted sensors to provide high definition images. These allow detection and inspection of lesions within luminal structures, such as the gastrointestinal (GI) tract and bronchus, enabling complex interventions to be performed (Shen et al., 2019). Some specialist procedures, such as inspection of the pancreatic and bile ducts, are carried out using a fibre optic imaging bundle (Fig. 2(f)) that transmits a low resolution image to a camera at the instrument's proximal end (Voaklander et al., 2016). The most common surgical optical imaging device outside of MIS is the operating microscope (Fig. 2(g)), and variations on its design are used for microsurgical tasks in neurosurgery, otolaryngology, ophthalmology, gynaecology, dentistry and plastic surgery (Kriss and Kriss, 1998; Uluç et al., 2009). Although primarily used by the surgeon for direct, magnified, vision of the operating field, many systems have additional ports that can be used to mount cameras or additional eyepieces (Hoerenz, 1980).

Standard colour cameras are chiefly used to relay images of the operating field to the surgeon, particularly in MIS or microsurgery. Therefore, beyond improvements in resolution, magnification and access to challenging anatomical sites, enhancement of the surgeon's ability to assess the viability of a tissue or characterise the extent of disease is limited to what would be observable under direct vision. As a result many decisions are still subjective (de Cunha et al., 2004) and heavily dependant on the experience of the operator (Ignjatovic et al., 2009). Surgery has increasingly been looking to advanced optical instrumentation to improve this by providing objective assessments of tissue health in real time. Some optical imaging modalities have gained clinical adoption, such as narrowband imaging (NBI), which uses a narrow range of green and blue wavelengths to generate high contrast images of blood vessels. This technology has had limited success in delivering on its diagnostic potential as subjective human interpretation is still required (Ignjatovic et al., 2009; Rees et al., 2016). Exogenous fluorescent contrast agents are also capable of identifying blood vessels and recent trials have indicated potential for detection of cancer (DSouza et al., 2016). Although several specialised agents with molecular specificity are undergoing *in vivo* trials (Nagaya et al., 2017), routine imaging capabilities are still restricted to a small number of clinically-approved dyes.

### 1.3 Spectral imaging

Spectral imaging techniques capture the reflectance spectrum of the tissue over an entire surface, assembling a *datacube* consisting of one spectral and two spatial dimensions (Fig. 1(c)). Depending on the number of bands acquired the imaging system may be termed multispectral (MSI; up to 10 s) or hyperspectral (HSI; up to 100 s). Lu and Fei (2014) describe the main approaches used to assemble the datacube, which may be broken down into *scanning* (either in the spatial or spectral dimensions) and *snapshot* (acquiring spatial and spectral information simultaneously) modalities. These spectral data can then be used to generate maps of tissue function (Tetschke et al., 2016; Kawauchi et al., 2019), structural abnormalities (Bedard et al., 2013) or enhanced contrast between different organs and structures (Akbari et al., 2008a, 2008b).

There are a variety of surgical imaging techniques with spectral sensitivity, each bringing particular advantages in depth penetration (photoacoustic tomography; (Zhu et al., 2018)), separation of absorption and scattering effects (spatial frequency domain imaging (SFDI); (Gioux et al., 2011)) and specificity (fluorescence guidance; (Zhou and El-Deiry, 2009)). Despite their advantages, these methods inevitably come with caveats related to hardware complexity, motion artefacts or the need for exogenous contrast agents. Although some of the demand for spectral imaging technology has been motivated by the increasing availability of fluorophores for diagnostics and guidance (Debie and Hernot, 2019), a detailed discussion of this field is outside the scope of this paper and relevant reviews may be found elsewhere (DSouza et al., 2016). In this review we concentrate on purely reflectance-based MSI and HSI implementations due to their increasing availability, compatibility with other clinical instruments, and the growing range and power of data processing tools.

A review by Lu and Fei (2014) summarised the principal hardware designs and general medical applications of spectral imaging. Other relevant reviews have also been published, focussing on specific medical applications such as neuroimaging (Giannoni et al., 2018), wound assessment (Thatcher et al., 2016) and gastroenterology (Ortega et al., 2019). However, the ongoing growth of this research field, coupled with increasing commercial interest, has provided the motivation to write this review. In this article we aim to:

• Focus on surgical applications with particular emphasis on MIS and MIS-compatible approaches
• Describe current clinically-approved commercial systems
• Discuss new computational methods including estimation of spectral detail from conventional colour images
• Discuss methods and data for training and validating imaging systems as well as for understanding fundamental variability across organs and patients.

Papers for this review were drawn from the Pubmed database, Google Scholar and arXiv with an emphasis on those published after spring 2013, which was the end of the period covered by Lu and Fei (2014). The search terms used were spectral imaging (including multi/hyperspectral), surgery (including minimally invasive, minimal access, and open) and *in vivo*.

In this paper we first outline the major developments in imaging hardware designs, separated by acquisition method, discussing their relative merits with reference to specific clinical applications. Commercial spectral imaging cameras are highlighted, including complete systems optimised for clinical use. Data analysis is covered in Section 3, beginning with a brief overview of light-tissue interaction theory and modelling before progressing to regression methods, classification problems and machine learning. Section 4 discusses methods of validating SSI systems, including computer simulations, tissue phantoms and real tissue (*ex vivo* and *in vivo*), and their ability to quantify performance. The discussion in Section 5 identifies the major challenges facing SSI technology in its push toward routine clinical use, and proposes ways the research community may focus their efforts to overcome them.

## 2 Surgical spectral imaging (SSI) hardware

The continued growth in spectral imaging activity has seen further increases in the application and scope of SSI methods, as evidenced by Table 1. Detailed descriptions of datacube acquisition mechanisms are described in other reviews, therefore we summarise the main types relevant to SSI (Sections 2.1 to 2.3), along with a discussion of practical and application-specific constraints.

**Table 1:** Comparison of recent SSI methods compatible with both open surgery and MIS. Datacube processing time, where available, is given in brackets beside the frame rate.

Author	Scan Method	Dispersive element	Spectral Range (nm)	Spectral Resolution (nm)	No. Bands	Spatial Resolution (Px)	Frame Rate (fps)	Application	Target Tissue
Hohmann et al. (2017)	Spectral	AOTF	400–650	12–20	6	350 × 370	2 (1.55 s)	Gastroscopy	Stomach
Halicek et al. (2017); Halicek et al. (2019)	Spectral	LCTF	450–900	7–20	91	1392 × 1040	0.02 (4 min)	Head and neck cancer *ex vivo*	Tongue, pharynx, larynx, mandible
Lu et al. (2015)	Spectral	LCTF	450–900	20	45–226	1392 × 1040	0.008 (1 min)	Small animal tumour	Various abdominal
Clancy et al. (2015)	Spectral	LCTF	460–690	7–20	24	1024 × 768	0.14 (45 s)	Laparoscopy	Bowel
Han et al. (2016)	Spectral	Filter wheel	405–665	10	27	582 × 752	0.24 (not given)	Colonoscopy	Bowel
King et al. (2015)	Spectral	Filter wheel	420–972	10	8	1392 × 1040	- (not given)	Open	Skin
Wirkert et al. (2016); Ayala et al. (2019)	Spectral	Filter wheel	470–700	20–25	8	1228 × 1029	2.5 (0.18 s)	Laparoscopy, open	Bowel, brain
Olweny et al. (2013)	Spectral	Tuneable source (DMD)	520–645	5	>100		3 (0.2 s)	Laparoscopy	Kidney
Kavvadias et al. (2013)	Spectral	Tuneable source (VOF)	400–1000	11–14	15	1920 × 1440	0.5 (not given)	Hysteroscopy	Endometrium
Bolton et al. (2018)	Spectral	Tuneable source (multi LED)	400–950	11–100	13	2592 × 1944	0.03 (not given)	Erythaema	Skin
Fawzy et al. (2015)	Spectral	Tuneable source (filter wheel)	400–760	15	18	659 × 494	15 (2.4 s)	Bronchoscopy	Lung
Wisotzky et al. (2018)	Spectral	Tuneable source (filter wheel)	400–700	20	16	1920 × 1080	- (not given)	Otolaryngology	Mastoid, parotid, gland
Ma et al. (2016a)	Spectral	Tuneable source (multi LED)	530–630	18–33	2	512 × 512	~10 (not given)	Neuroimaging	Brain
Luthman et al. (2018)	Snapshot	Mosaic sensor	470–630 600–1000	<15	16 (VIS) 25 (NIR)	512 × 256 409 × 218	90 (10 min)	Gastroscopy	Oesophagus
Wirkert et al. (2018)	Snapshot	Mosaic sensor	470–630	<15	16	512 × 256	90 (7.5 ms)	Laparoscopy	Kidney
Nishidate et al. (2013); Akter et al. (2017)	Snapshot	RGB	450–690	–	25	1024 × 768	15 (~5 s)	Open	Skin, liver
Jones et al. (2017b)	Snapshot	RGB	500–620	–	13	1024 × 768	30 (33 ms)	Laparoscopy	Bowel
Clancy et al. (2014)	Snapshot	RGB	470–635	25/60	3	1024 × 768	7 (~3 ms)	Laparoscopy	Bladder, bowel
Kester et al. (2011); Bedard et al. (2013); Shadfan et al. (2017)	Snapshot	IMS	480–656	4–10	8–60	200 × 200	8–10 (not given)	Gastroscopy	Oesophagus
Lin et al. (2018b)	Snapshot	Fibre bundle + spectrograph	460–690	6	24	1024 × 768	2 (500 ms)	Laparoscopy, otolaryngology	Bowel, larynx
Khoobehi et al. (2012); Khoobehi et al. (2014)	Snapshot	Fibre bundle + spectrograph	–	1	–	458 fibres (~22 × 22)		Fundus camera	Optic nerve head
Clancy et al. (2016b)	Spatial	Spectrograph	350–750	5	>200	1000 × 50	0.5 (not given)	Colonoscopy	Bowel
Kiyotoki et al. (2013); Mori et al. (2014); Kumashiro et al. (2016)	Spatial	Spectrograph	400–800	5–10	72	640 × 480	0.06–0.2 (~10–90 s)	Colonoscopy, neurosurgery, gastroscopy	Stomach, brain, bowel
Tetschke et al. (2016)	Spatial	Spectrograph	500–1000	5	100–750	640 × 480	0.1 (40 s)	Machine perfusion	Kidney

### 2.1 Spectral scanning

Spectral scanning methods sequentially acquire images at different wavelengths. One common approach is to flood the scene with broadband illumination and include a bandpass filter in front of a monochrome camera to image the reflected light. A different wavelength is acquired by mechanically switching using a filter wheel (Fig. 2(a)) (King et al., 2015; Wirkert et al., 2016; Clancy et al., 2018), or by using an electronically-tuneable device such as a liquid crystal tuneable filter (LCTF) or acousto-optical tuneable filter (AOTF). This has the advantage of being compatible with currently-used high-brightness xenon surgical light sources, while the relatively simple detection hardware can be connected directly to clinical optical imaging instruments as the transmission filter is the only addition to the beam path. Therefore, with minimal modification, filter-based SSI devices can be attached to rigid endoscopes (Arnold et al., 2011; Kavvadias et al., 2013; Clancy et al., 2015) and operating microscopes (Ma et al., 2016a), or positioned on an articulated arm for open or external imaging procedures (Orfanoudaki et al., 2005; Holmer et al., 2018; Kulcke et al., 2018). Relative tissue-camera motion may cause artefacts in the datacube, particularly during long sequences, although this may be rectified post-acquisition using registration techniques (Clancy et al., 2012b; Du et al., 2015).

Scanning may also be implemented in illumination, using a white light source and monochromator (Regeling et al., 2016), filter wheel (Gu et al., 2016; Han et al., 2016; Wisotzky et al., 2018), variable optical filter (VOF) (Kavvadias et al., 2013), AOTF (Hohmann et al., 2017) or digital micromirror device (DMD; Fig. 2(b)) (Koh et al., 2009; Zuzak et al., 2011). Triple bandpass illumination filters overlapping with RGB detection can be used to achieve a degree of parallelisation and higher acquisition rates (Fawzy et al., 2015). The increasing availability of high-power light-emitting diodes (LEDs) at multiple wavelengths has enabled creation of light-efficient MSI sources capable of switching at high speed (Guevara et al., 2013; Bélanger et al., 2016) and at low-cost (Bolton et al., 2018). With a high quantum efficiency sensor, such as an electron-multiplying charge-coupled device (EMCCD) or scientific complementary metal oxide semiconductor (sCMOS), a strobed LED source can also incorporate fluorescence excitation along with reflectance measurements, as demonstrated by Ma et al. (2016a). This allows quantification of haemodynamic events, which can further be used to correct the observed fluorescence signal (Ma et al., 2016b). The disadvantages of LEDs are inefficient fibre-coupling, which is problematic for MIS applications, and inconsistent spectral resolution across the visible and near-infrared range. Low-loss multiplexing using light guides is possible (Clancy et al., 2012a) but is difficult to scale to a large number of wavelengths. The challenge for practical implementation of tuneable light source-enabled SSI is the need to achieve fast electronic or software-enabled synchronisation with the camera, which requires interfaces that are not typically available on standard clinical MIS or tip-mounted endoscopic cameras. In open surgery cases, ambient light is an added complication as there is no rejection of the out-of-band signal, nor can the spectrum of the background be measured. This reduces the overall signal-to-noise ratio (SNR) to a greater degree than in systems where scanning is accomplished on the detection side and ambient light may be accounted for during system spectral correction.

### 2.2 Spatial scanning

Spatial scanning involves acquiring the spectral information from a single point (whisk-broom (Qiu et al., 2010)) or line (push-broom, Fig. 2(c); (Khoobehi et al., 2004; Clancy et al., 2016b; Tetschke et al., 2016)) and scanning across the field-of-view using galvanometer (galvo) mirrors or robotic actuation mechanisms (Avila-Rencoret et al., 2015). Spectrometers and imaging spectrographs enable high spectral resolution (a few nm, band full-width at half-maximum (FWHM)) at hundreds of wavelengths. Spatial resolution is limited to the number of scan lines acquired and the constraints imposed by any motion in the target tissue. The necessity to house multiple dispersive optical elements, particularly in the case of pushbroom imagers, has made miniaturisation of these systems a challenge. However, developments such as sensor-mounted Fabry-Pérot interference filters, have enabled the manufacture of hyperspectral devices with spectral sensitivity integrated along one dimension of the sensor (Pichette et al., 2017). This means that the system footprint is limited by the scanning mechanism employed.

The main limitation to surgical application of this method is its sensitivity to motion artefacts and the difficulty in aligning the spatial slices post acquisition. *In vivo* imaging of internal anatomy using flexible endoscopes has been demonstrated with this technique (Clancy et al., 2016b), with scan times minimised to limit the effect of motion and deformation during data capture (Kumashiro et al., 2016). Alternatively, simultaneous wide-field imaging using a second camera can allow motion-correction (Yoon et al., 2019a). External anatomy and more rigid internal organs, less prone to gross motion and deformation have yielded high resolution results in wound-healing (Calin et al., 2015; Holmer et al., 2016), neuroimaging (Mori et al., 2014; Fabelo et al., 2018) and flap transplantation monitoring (Kulcke et al., 2018).

### 2.3 Snapshot acquisition

Snapshot imagers capture all three dimensions of the datacube simultaneously. This is usually done by distributing both spatial and spectral information across a single image sensor. A comprehensive review of snapshot spectral imaging devices and a detailed treatment of their operating principles is presented by Hagen and Kudenov (2013). Their principal disadvantage is that fast acquisition is achieved by compromising on spatial resolution. Depending on the mechanism used, this effectively limits the number of wavebands to the MSI domain. For example, for a 9-band snapshot imager based on a 2048 × 1088 sensor, the final spatial resolution will be 0.25 MP, but for 25 bands this drops to 0.09 MP. This approach has found particular use in applications where the sensor size is not the limiting factor for spatial resolution, such as fibrescopes. (Shadfan et al., 2017). Improved spectral performance can be achieved using fibre bundles to map spatial locations to inputs on a spectrograph (Khoobehi et al., 2012), while spatial resolution can be improved through combination with simultaneous RGB imaging (Lin et al., 2018b).

Snapshot detectors using multiple optical elements, such as prisms and image-slicing mirrors, may add significant weight through glass components and metal enclosures, which constrains their use. Their complex bespoke design further limits the range of applications in which they can be used. For these reasons snapshot imagers have remained relatively inaccessible for general research applications until recently, with the advent of spectral imaging sensors. A number of companies now offer products in this area, with spectral filters positioned directly onto the sensor. These are configured as *tiled*, consisting of an array of relatively large filters covering 1000s of pixels, and *mosaic*, having a repeating pixel-level pattern over the entire sensor, designs (Fig. 2(d)). They are currently available both in OEM form and as part of a complete camera package (Pichette et al., 2016). Like the spectral scanning systems, discussed in Section 2.1, these cameras can be placed directly in place of existing clinical digital cameras on rigid endoscopes (Wirkert et al., 2018), fibrescopes (Luthman et al., 2018), and operating microscopes.

### 2.4 Applications and system choice

The hardware configuration to be used for a particular SSI application must be made with an appreciation of the advantages and disadvantages of each approach and is a question that cannot be answered by a single metric. Variable filters such as the LCTF and AOTF offer the advantages of being free from moving parts, having electronic control and flexibility in wavelength choice. However, the devices are optically inefficient: for example, LCTFs optical transmittance ranges from 5% at 420 nm to approximately 35% at 700 nm (CRi, 2019; Thorlabs, 2019), necessitating long camera integration times or sophisticated high-sensitivity sensors to collect enough light. Cameras employing sCMOS, intensifiers or EMCCDs (Martin et al., 2006; Arnold et al., 2010) have been used in these systems to operate at speeds approaching video rates. Due to the sequential nature of the acquisition process, the datacube may be subject to artefacts induced by cardiac, peristaltic or respiratory motion. This can cause significant misalignment in the image stack, particularly if a large number of wavelengths are required, resulting in errors in the spectra recorded at particular spatial points. These errors can be corrected using a separate colour camera to track motion and apply corresponding adjustments to the spectral channel *via* photogrammetry techniques (Clancy et al., 2012b). Alternatively, computer vision tools for non-rigid registration can be used to align the spectral images using contrast and intensity-based features (Stoyanov et al., 2012; Du et al., 2015).

Pushbroom hyperspectral sensors are desirable for applications where high spectral resolution is required. For example, to achieve spectral unmixing of multiple absorbers and scattering contributors (Randeberg et al., 2010) or classify lesions (Kumashiro et al., 2016). Pushbroom HSI cameras offer great application versatility as they provide high resolution data at hundreds of wavelengths. One of the main practical drawbacks of these devices for surgical use is that they are much more sensitive to motion, lacking spatial cues in individual scan lines, rendering vision-based registration techniques unsuitable. There are practical limitations to mounting these cameras on surgical imaging equipment. Scanning mechanisms need to be mounted with imaging spectrographs, adding weight, complexity and the potential for misalignment. Furthermore, even the most compact spectrograph designs are relatively large even without the imaging sensor, which would prevent their use in hand-held devices such as laparoscopes (typical MIS cameras weigh <100 g). New systems, such as the Snapscan (IMEC, Belgium) aim to counter these limitations by integrating scanning, spectral dispersion and detection in a single unit. Although a much more robust design, its weight (~500 g) remains a factor when considering mechanical constraints.

The footprint of snapshot devices, particularly sensors of the type shown in Fig. 2(d), is comparable to conventional cameras already used clinically and the design is mechanically robust. The strength of this method in surgery is that it is immune to motion-induced misalignments, thus enabling capture of fast processes and delivery of functional information to the clinician in real-time. These devices have been demonstrated in neurosurgery (Pichette et al., 2016), flexible endoscopy (Luthman et al., 2018; Wang et al., 2018), and retinal imaging (Firn and Khoobehi, 2015). Nevertheless, there remains some important considerations when considering a sensor of this type. Snapshot acquisition requires the 3D datacube to be distributed on a 2D sensor, meaning that capture speed comes at the expense of spatial resolution and/or the number of available wavebands. The type of filter used by the sensor should also be considered when choosing an application-specific imager. Signal cross-talk between adjacent pixels may become significant in mosaic-type sensors, where pixel-level filters are used, leading to further degradation of spatial resolution. In the case of interference filters, the transmission bandwidth and centre wavelength is dependant on the distribution of angles of incoming light (Frey et al., 2015). Therefore, careful characterisation of the imaging optics is needed, with the final spectral resolution determined by the ratio of the camera focal length to the diameter of the entrance pupil (the system's F-number). This may vary significantly between surgical scenarios and the optical instruments shown in Fig. 2 (e–h). Interpretation of signals from these pixel-level sensors is also complicated by the spectral response of the filters themselves, with some containing prominent side-lobes and secondary passbands, which result in significant cross-talk between the blue and red ends of the spectrum (Wirkert et al., 2018; Wisotzky et al., 2019).

Recognising the trade-offs that must be made between spatial, spectral and temporal resolution, and bound by experimental constraints, many researchers have adopted a two-stage development process, using high spectral resolution HSI scanning devices for initial exploratory work and then proceeding to more streamlined and efficient MSI systems. This necessitates data reduction techniques to isolate the spectral bands in HSI datacubes that contain the most clinically-significant information without compromising on specificity or sensitivity. Wirkert et al. (2014) analysed surgical datacubes comprised of 30 wavelengths, captured using an LCTF-based system, and used an information theory-based approach to identify eight optimal bands for oxygen saturation estimation. This meant that the imaging hardware could be switched to a fast filter-wheel platform. A similar approach was taken by (Kiyotoki et al., 2013) to identify five bands that would optimally discriminate between adenomatous and normal tissue in the colon. Reducing the number of wavebands brings several advantages, including reducing the data storage burden, enabling use of high throughput transmission filters, and limiting the impact of tissue motion through faster datacube acquisition time.

Given the dynamic nature of the operating theatre, speed remains an important performance metric of surgical imaging systems in general. Of the SSI systems surveyed here and listed in Table 1 it is unsurprising that the snapshot methods boast the highest acquisition rates. Those based on RGB camera hardware can typically operate at 30 fps while more recently-developed tiled/mosaic sensors can acquire at up to 90 fps. Both spatial and spectral scanning methods are considerably slower, with most achieving framerates of less than 0.5 fps. Just one of the cases reported speeds approaching video rate, using an optimised filter wheel setup running at 15 fps (Fawzy et al., 2015). The next closest to this used fast-switching light sources (Hohmann et al., 2011; Olweny et al., 2013; Ma et al., 2016a) and filter wheels (Wirkert et al., 2016). The most significant remaining bottleneck though, is the processing method used. The computation times associated with each of the techniques is less than 1 fps in most cases, with the 133 fps convolutional neural network (CNN)-based method quoted by Wirkert et al. (2018) standing alone as a truly videorate/realtime example. Even allowing for the wide variety of processing techniques, datacube sizes, computer hardware and implementation it seems clear that handling these datasets remains a challenge. With this in mind it is worth considering what ‘realtime’ means in the context of the surgical application before pursuing speed over other performance metrics. For example, a single processed image showing a map of oxygenation or perfusion, processed in a couple of seconds, may be just as clinically valuable as a continuous, realtime measure of the same scene. Conversely, an SSI-supported vision system to identify early-stage precancerous lesions during exploratory procedures would have much higher temporal demands.

### 2.5 Commercial systems and clinical translation

Commercial activity in spectral imaging devices has increased steadily over the past decade and there are now several options from OEM-scale sensors to complete systems spanning a range of application areas. Table 2 summarises the range of currently-available systems, along with references to research articles in which they have been cited. Perhaps the most notable recent development is the continued refinement of snapshot sensors, which have dramatically reduced the hardware footprint and increased acquisition speed, both prerequisites for surgical use. As this technology begins the mature the variety of sensors has grown. The Ocean Insight (formerly Ocean Optics/Pixelteq) PixelCam can deliver six bands in the visible range, while IMEC have a range of tiled and mosaic sensors available from different camera manufacturers (Ximea, PhotonFocus) spanning visible and near-infrared wavelengths. The CMS range from Silios Technologies includes mosaic sensors comprising eight filtered and one monochrome (i.e., unfiltered) channel.

**Table 2:** Commercial spectral imaging devices and systems for research and clinical use.

Manufacturer	Model	Spectral resolution (nm)	Spatial Resolution	Bands	Spectral Range (nm)	Datacube Acquisition Time (s)	Citing articles
Diaspective Vision GmbH	TIVITA (formerly TI-CAM)	5	640 × 480	100–750	500–1000	5	Holmer et al. (2016); Tetschke et al. (2016); Holmer et al. (2018); Kulcke et al. (2018); Jansen-Winkeln et al., 2019; Köhler et al., 2019
Hypermed, Inc.	HyperView/OxyVu	^‡^	^‡^	8	400–700	<1	Yudovsky et al. (2011)
Kent Imaging, Inc.	Snapshot NIR	^‡^	^‡^	4	670–940	<1*	Hartwig et al. (2016)
IMEC	CMV2K-SSM4 × 4–9.2.10.3 Ximea xiQ	15	512 × 256	16	470–630	0.01–1	Luthman et al. (2018); Wirkert et al. (2018)
IMEC	SSM5 × 5 5.4.20.8 Ximea xiQ	15	409 × 218	25	600–1000	0.01–1	Luthman et al. (2018)
Optronic Laboratories	OL-490	5	NA^ǁ^	>100	380–780	NA^ǁ^	Zuzak et al. (2011); Olweny et al. (2013)
Ocean Insight (Pixelteq)	PixelCam	60	2048 × 2048	6	400–1000	0.067	(Vemuri et al., 2019)
Ocean Insight (Pixelteq)	SpectroCam	10–100	2456 × 2058	8	400–1000	0.27	Clancy et al. (2013); Moccia et al. (2018); Wirkert et al. (2018)
Ocean Insight (Fluxdata)	FD-1665-MS	50	1628 × 1236	3–7	400–1000	0.01	Sohaib and Robles-Kelly (2015)
Photonfocus	MV1-D2014 × 1088-HS03	15	2048 × 1088	16	470–630	0.02	Wang et al. (2018)
Photonfocus	MV1-D2048 × 1088-HS05-G2	10–12	2048 × 1088	150	470–900	42 fps^†^	
Norsk Elektro Optikk	HySpex VNIR 1800	3.26	1800	182	400–1000	260 fps^†^	Bjorgan and Randeberg (2015); Paluchowski et al. (2016)
IMEC	Snapscan NIR Snapscan VNIR	10–15	3650 × 2048	>100 >150	600–970 470–900	0.2	
IMEC	Linescan NIR Linescan VNIR	<10	2048	>100 >150	600–1000 470–900	2720 lines/s^†^	
Silios Technologies	CMS-C CMS-V CMS-S	40	426 × 339	8	500–830 650–930	0.02	Waterhouse et al. (2017)
Perkin Elmer	Nuance/Maestro	7–20	1392 × 1040	500	450–950	5 (16 bands)^§^	Lu et al. (2015); Halicek et al. (2017); Halicek et al. (2019)
Surface Optics	Light Shift	25	512 × 512	16	400–1000	0.0333	

*Includes haemoglobin estimation processing. ^†^Acquisition rate for individual spatial line. Needs line-scanning mechanism for image formation. ^‡^Information not available. ^§^dependant on number of bands and camera integration time. ^ǁ^System is a stand-alone multispectral light source; camera/detector is user-dependant.

Commercial spectral scanning devices using tuneable light sources are available from Optronic Laboratories (OL-490), but scanning in detection is more common, with LCTF (Perkin Elmer Nuance/Maestro) and filter wheel options (Ocean Insight SpectroCam). Pushbroom scanning imagers, like the Hyspex (Norsk Elektro-Optikk), have been most commonly-used in skin imaging, where there is a greater degree of control over potential tissue-camera motion.

**Fig. 3:**
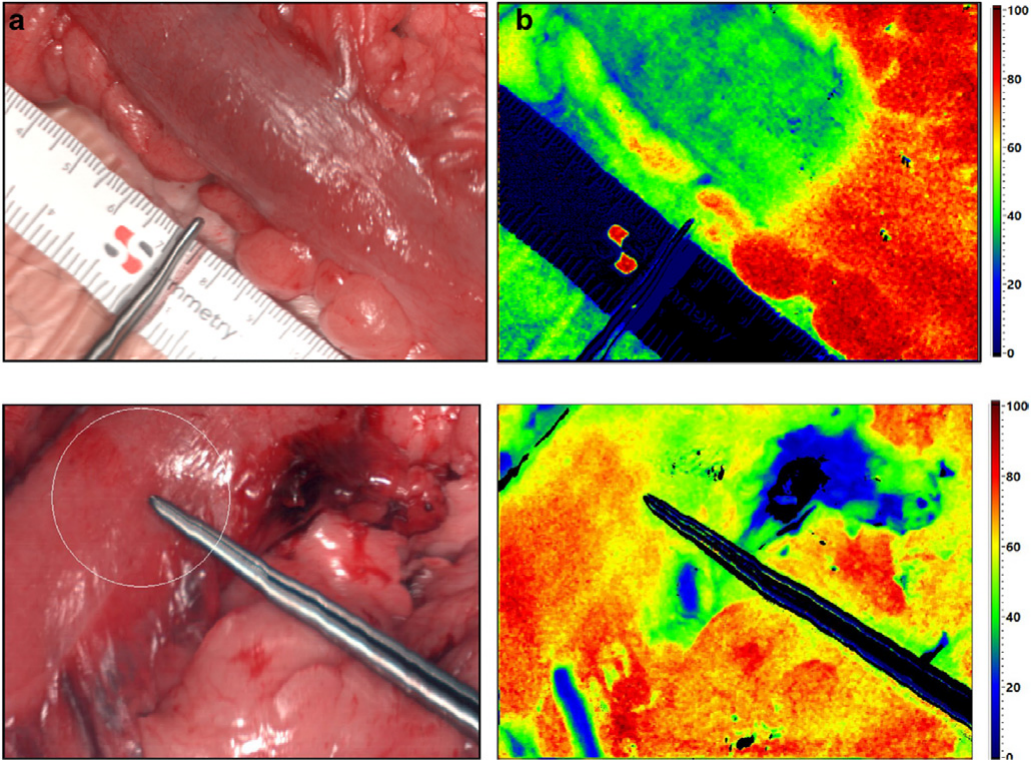
Top: Intraoperative oxygenation mapping during transection of the large bowel. Maps of SO_2_ show a well-defined border between perfused and non-perfused tissue after separation of the marginal artery. The location of this border differs from that chosen by a surgeon, indicated by the steel instrument, following inspection. See Table 2 for acquisition details. Image taken from Jansen-Winkeln et al., 2019, Reprinted by permission from Springer Nature Customer Service Centre GmbH: Springer International Journal of Colorectal Disease (Determination of the transection margin during colorectal resection with hyperspectral imaging (HSI), Boris Jansen-Winkeln et al.)© (2019). Bottom: choosing a site to create an oesophagogastric anastomosis, with SO_2_ indicating a well-vascularised region. Image taken from (Köhler et al., 2019). Reprinted by permission from Springer Nature Customer Service Centre GmbH: Springer Surgical Endoscopy (Evaluation of hyperspectral imaging (HSI) for the measurement of ischaemic conditioning effects of the gastric conduit during esophagectomy, Hannes Köhler et al.)© (2019).

Clinical translation remains limited, with few systems on the market for human use. The hyperspectral TIVITA series imagers (Diaspective Vision GmbH, Germany) have options for general tissue imaging, wound-healing and surgery, and have demonstrated potential utility in gastric anastomoses (Köhler et al., 2019) and colorectal transection margins (Jansen-Winkeln et al., 2019) (Fig. 3). The multispectral HyperView (Hypermed Imaging, Inc., USA) and Snapshot NIR (Kent Imaging, Inc., Canada) are handheld devices for perfusion and oximetry (Hartwig et al., 2016). An earlier iteration of the HyperView system, the OxyVu, was also used to characterise burn depth in animal models (Chin et al., 2016). Both can generate maps of relative haemoglobin concentration and oxygen saturation, while the TIVITA system provides additional indices relating to perfusion and water content. Complete systems for small animal imaging *in vivo*, such as the Maestro (Perkin Elmer, Inc., USA), have been used to collect spectral imaging data from head-and-neck cancer xenografts (Lu et al., 2015; Halicek et al., 2019).

## 3 Spectral image analysis

After acquisition, the most fundamental processing step that is carried out on the datacube is to convert the reflected intensity measurements to reflectance spectra by correcting for the system's spectral sensitivity, normally achieved using a reference reflectance standard measurement (Lu and Fei, 2014). These spectra can be further converted to absorbence by taking the negative logarithm of reflectance. Interpretation of these results and quantification of tissue properties can then be achieved using light-tissue interaction models (Section 3.1) or data science techniques (Section 3.2).

### 3.1 Light propagation models for surgery

If it is assumed that the forward process of light propagation is well-described by mathematical models or simulations then the inverse problem may yield the tissue properties of interest from spectral measurements. An approximation of the total absorption coefficient of a tissue (*μ_a,tissue_*) under investigation can be calculated, using Eq. (1), as a linear sum of the contributors described in Section 1.1:

(1) $$\mu_{a,tissue}=\varepsilon_{Hb}c_{Hb}+\varepsilon_{HbO_{2}}c_{HbO_{2}}+\varepsilon_{\text{water}}c_{\text{water}}+\varepsilon_{fat}c_{fat}+\varepsilon_{bili}c_{bili}$$

where *c* [M or mol cm^−3^] and *ε* [*M*^−1^ cm^−1^] represent the concentration and specific molar extinction coefficient, respectively, of deoxyhaemoglobin (*Hb*), oxyhaemoglobin (*HbO_2_*), water, lipids (*fat*) and bilirubin (*bili*). Jacques (2013) has compiled a set of numerical values and typical volume fractions for various organs that can be used with Eq. (1) to generate a realistic tissue absorption spectrum. Losses due to scattering from the continuum of particle sizes may be described by Mie scattering theory, which assumes spherical scatterers and generally predicts a smooth decrease with increasing wavelength. Subcellular particles having diameters smaller than the wavelength of light are subject to the Rayleigh limit (Tuchin, 2015b). Therefore, the observed reduced scattering coefficient (*μ_s_’*) is a combination of both regimes and is often described by the empirical model shown in Eq. (2) (Hidović-Rowe and Claridge, 2005; Jacques, 2013; Mourant et al., 2014; Pichette et al., 2016):

(2) $$\mu_{s}^{\prime }=a\lambda^{-b}$$

where *λ* is wavelength, *a* is a scaling factor and *b* is termed the *scattering power*, indicating the strength of the effect.

A common method to obtain the absorption and scattering coefficients from reflectance data is to perform iterative fitting on a forward model based on diffusion theory (Spott et al., 1998). This has been used successfully with point probes in the pancreas to identify adenocarcinomas and pancreatitis (Wilson et al., 2009). This was expanded to HSI of the skin by Randeberg et al. (2010) and allows calculation of relative concentrations of absorbers, scatterer size and distribution, and estimation of blood vessel density (Randeberg et al., 2005). This is a computationally-expensive process for imaging, given the complexity of the model and number of free variables, to solve the inverse problem iteratively and requires more advanced parallel computing methods (Bjorgan and Randeberg, 2015).

A simpler approach uses the modified Beer-Lambert law (Villringer and Chance, 1997), which states that absorbence (*A*) is proportional to the concentration (*c*) of the light-absorbing compound and the distance that the light travels through the tissue (*L*). The constant of proportionality is the extinction coefficient (*ε*) and there is an offset term (*G*) to account for scattering losses, approximated as being wavelength-independent (Eq. (3)):

(3) $$A\left(\lambda \right)=Lc\varepsilon \left(\lambda \right)+G;\mu_{a}=c\varepsilon$$

A differential pathlength correction factor (*DPF*), determined experimentally (Pichette et al., 2016), or using computer simulations (Hillman, 2007), may also be included to correct wavelength-dependant variations in *L*. To simplify calculations for SSI an ‘equal pathlength’ assumption can be made, and the *DPF* and *L* incorporated with *c*, which becomes a *relative concentration*.

If *A* is measured using the SSI device, and *ε* is known *a priori*, then Eq. (3) can be solved using linear least squares regression to compute the relative concentrations and *G*. Subsequent calculation of total haemoglobin (*THb* = *c*_*HbO2*_ + *c_Hb_*) and oxygen saturation (*SO_2_* = *c_HbO2_*/*THb*), can then be easily achieved. Eq. (3) can be solved analytically, making it attractive for fast processing over an entire image, and has been used to quantify haemoglobin and perfusion-related variables in the heart (Nighswander-Rempel et al., 2003), bowel (Clancy et al., 2015), uterus (Clancy et al., 2016a), skin (Zuzak et al., 2002) and mammary carcinomas (Sorg et al., 2005). Calculations can also be significantly simplified if some experimental constraints are applied. If temporal changes are relevant, in a study of brain activity to various stimuli, for example, then SSI data acquired from the same stationary tissue area can be analysed to calculate changes in chromophore concentration, as the pathlength terms cancel out (Bouchard et al., 2009).

Computer simulations of photon propagation in tissue can be used to form a forward model, avoiding some of the assumptions made by diffusion theory and Beer-Lambert. Claridge and Hidović-Rowe (2014) have developed an image analysis method based on a multilayer Monte Carlo (MC) model for examining pathological changes in the mucosa of bowel tissue *ex vivo*. The model is used to simulate reflectance spectra for ranges of tissue optical properties. These spectra are fitted to experimental results in a fast iterative process that uses the Kubelka-Munk approximation of diffuse reflectance as part of the optimisation process (Hidović and Rowe, 2004; Hidović-Rowe and Claridge, 2005; Claridge et al., 2007). Wirkert et al. (2016) also employed a forward Monte Carlo model to estimate the tissue properties. In this case, multiple simulations for varying tissue oxygenation and blood volume conditions were used to train a random forest regressor and achieve rapid processing of high-resolution multispectral images during MIS (imaging and regression times of 400 ms and 180 ms, respectively).

### 3.2 Multivariate regression and classification algorithms

The previous section included an outline of analytical and linear regression methods used to unmix individual spectral contributors to the observed reflectance signal based on some assumptions and *a priori* knowledge of the principal chromophores. However, a purely data-driven method using a statistical model treating spectral data as predictors can avoid the assumptions of physical models. These are well-suited for imaging applications due to their computational efficiency and ability to detect subtle differences between classes. This is an attractive proposition in surgical segmentation problems, where lesions or anatomical structures of interest may have broadly similar reflectance characteristics to background tissue.

The review by Lu and Fei (2014) contains an overview of many of the most commonly-used statistical analysis methods applied to medical spectral images in general. For HSI this can include an initial dimensionality-reduction step, achieved using principal component analysis (PCA), minimum noise fraction (MNF) or independent component analysis (ICA), to extract the most information-rich spectral features and reduce redundancy in the data. These techniques transform the data into a subspace where it is arranged according to the amount of variance, thus enabling separation of different contributors to the signal, such as tumourous and healthy tissue (Lu et al., 2014; Chung et al., 2016). In addition to identification of diagnostic signals, this type of approach can also be used to reduce noise in the datacube by keeping only the transformed bands with high signal-to-noise ratio (Bjorgan and Randeberg, 2015). Dimensionality reduction helps to avoid overfitting problems associated with unsupervised methods such as k-means clustering, when used as a pre-processing step (Torti et al., 2018). K-means, which iteratively assigns pixels to ‘cluster centres’ by minimising their Euclidian distance, has been used to identify cancer of the breast (Khouj et al., 2018), colon (Baltussen et al., 2019) and brain (Torti et al., 2018), as well as hyperplasia in the endometrium (Kavvadias et al., 2013).

**Fig. 4:**
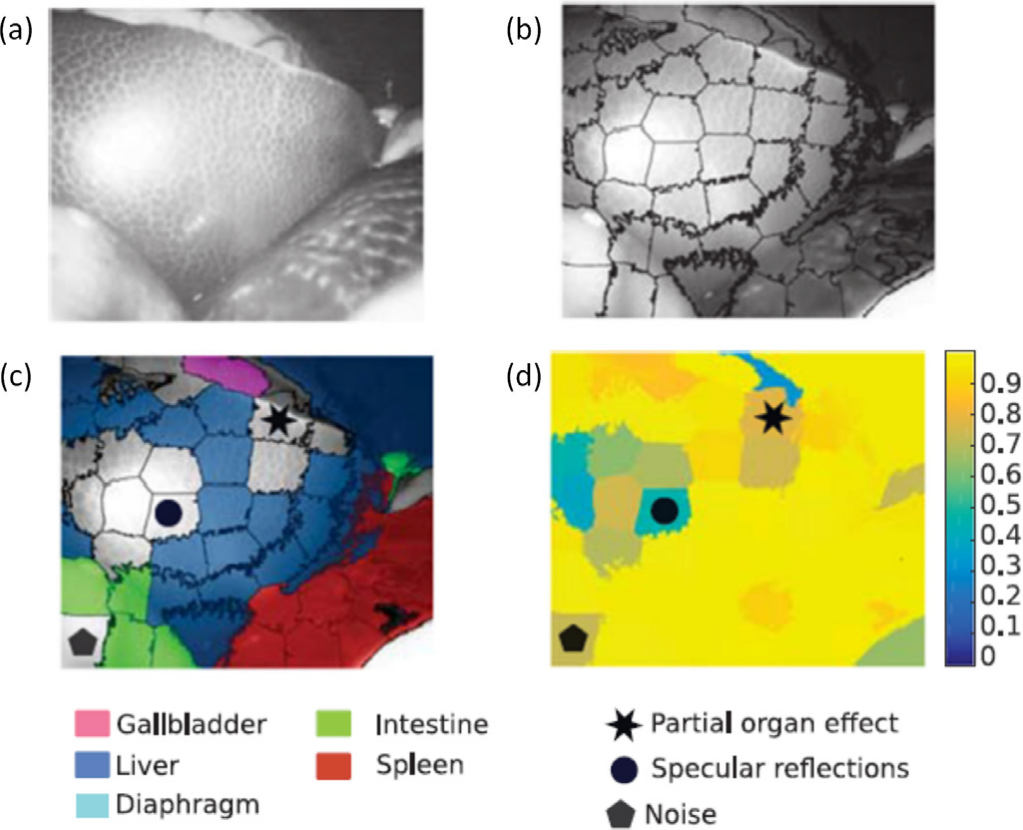
Improving classification accuracy through awareness of uncertainty in the data. (a) Spectral input image. (b) Input image after creation of texture-based superpixels. (c) Classification result showing only high-accuracy superpixels, as identified in (d) confidence map. See Table 2 for acquisition details. Adapted from Moccia et al. (2018).© 2018 IEEE. Reprinted, with permission, from Sara Moccia et al., Uncertainty-aware organ classification for surgical data science applications in laparoscopy. IEEE Trans. Biomed. Eng. 65 (11), 2649–2659.

Examples of supervised classifiers that have been used with SSI data are support vector machines (SVM) and spectral angle mapping (SAM). An advantage of SVMs is that they are robust to noisy, high-dimensional data, thus not requiring a feature selection step (Camps-Valls and Bruzzone, 2005). Akbari et al. (2012) used an SVM to classify cancerous and normal tissue in lung and lymph tissue histology slides, achieving 93–96% sensitivity and 98% specificity, while Hohmann et al. (2017) reported 57–66% sensitivity and 52–62% specificity for fresh tissue samples of gastric adenomas. Han et al. (2016) conducted an *in vivo* colonoscopy study with a five-band MSI system and classified adenomas with 95% sensitivity and 89% specificity. Spectral features can also be combined with measures of local texture to improve classification accuracy of SVM (Zhang et al., 2016). Intrinsic measures of uncertainty, using the Gini coefficient, have demonstrated further improvements in performance (Fig. 4) by identifying and excluding low accuracy superpixel subregions (Moccia et al., 2018). This allows automatic tagging of tissues *in vivo* with 96% accuracy when used with an 8-band multispectral datacube. SAM treats individual spectra as high-dimensional vectors, differentiating regions or tissue types using the angle between them. This has the advantage of being robust to variations in illumination strength or shadows (Martin et al., 2012).

Artificial neural networks (ANNs) and, more specifically, deep learning with CNNs have recently seen growth in detection, classification and segmentation problems in a variety of medical imaging modalities due to their efficient architecture and use of local context (Shen et al., 2017). Recent applications in spectral imaging analysis include use of CNNs to differentiate abdominal organs (Akbari et al., 2008b), and detect gastric (Hu et al., 2019) and head-and-neck cancers (Halicek et al., 2017, 2019). Reported accuracy in these studies is high (>95%) and a recent comparative study also suggests that CNNs may out-perform competing supervised classification methods, such as SVMs (Halicek et al., 2017). CNNs can incorporate multiple processing steps within the overall network architecture, including pre-processing calibration steps, within the network, which optimises speed when compared to other regressors such as random forests (Ayala et al., 2019). By avoiding more tailored regression approaches, CNNs may capture subtle signal variations that support interpretation or classification much more effectively. This would be logical given the performance increases demonstrated through deep learning in general computer vision and signal analysis problems.

The main limitation of these machine learning methods in general is that accuracy depends on the available labelled training data. This is a problem in surgical imaging, where a well-defined gold standard is lacking, and inter and intrapatient variability is large but the quantity of datasets is extremely limited. Common solutions used to boost training data are to use transformations (e.g., rotation, translation), pretrained networks and to use image patches (Shen et al., 2017). Previous work in other medical imaging modalities has also combined separate datasets in an effort to better represent variability across patients (Shin et al., 2016). Transfer learning, where a pre-trained network is adapted for use in a new setting, is a potential solution, and has already seen use in other medical image analysis problems, where available training data are insufficient (Signoroni et al., 2019).Wirkert et al. (2017) have recently demonstrated the potential of using this technique to adapt a network, trained on a generic simulated tissue, to unlabelled *in vivo* datasets. Recent efforts in developing unsupervised learning may translate to SSI problems and additional potential could be explored by training on phantom or controlled environments prior to fine tuning networks using small datasets of clinical data. In histopathology some recent efforts have shown that networks could capture information typically extracted through contrast agents and staining (Pei et al., 2019; Talo, 2019) and a similar methodology could be pursued in SSI.

Another challenge related to variability, and inherent in the ambiguous nature of the problem, is the fact that the mapping between measured optical signals and estimated physiological variables may not be one-to-one. To address this problem Ardizzone et al. (2019) has proposed invertible neural networks (INNs), which aim to learn the posterior probability distribution and represent ambiguity in the solution. This has recently been applied to MSI imaging of the brain (Adler et al., 2019).

### 3.3 Estimating spectral information from RGB

Deducing the underlying spectral properties of tissue using standard colour cameras would be a convenient way to realise an SSI system. The challenge here is that the inverse problem is ill-posed, with many different combinations of component spectra capable of producing a given RGB response. Regression models between MC-simulated spectra and observed RGB values enable calculation of relative concentrations of HbO_2_, Hb and melanin (Nishidate et al., 2008, 2011). Their accuracy, however, is subject to the MC model being a good match to reality. Variation in tissue layer thickness and/or scattering properties are not accounted for and contribute to errors in the estimated concentration values. Wiener estimation, alternatively, predicts the reflectance spectrum of an object using *a priori* knowledge of the camera's spectral sensitivity and the reflectance spectrum of the object under test (Stigell et al., 2007). This has been used to estimate melanin and haemoglobin concentrations in skin (Nishidate et al., 2013), scattering variables in the brain (Yoshida et al., 2015; Hasnat et al., 2016) and bowel oxygen saturation (Jones et al., 2016). Lin et al. (2017) demonstrated that a CNN could be used to estimate fine spectral data from RGB laparoscopic images. Strong qualitative agreement with reference MSI data was shown, although large errors at some wavelengths were noted. Subsequent work improved accuracy by adding sparsely-sampled high spectral resolution data to update and refine the CNN result (Lin et al., 2018b). Li et al., 2019 has modified this approach using a conditional generative adversarial network (cGAN) to bypass the spectral estimation step and generate maps of oxygen saturation directly.

## 4 Data assessment and validation

A clear validation process is necessary to understand the capabilities of a given SSI system, quantify its accuracy and understand variability across different hardware configurations and clinical settings. Validation data may be obtained from experiments using computer simulation, tissue phantoms, resected tissue, or *in vivo* measurements.

### 4.1 In silico

Monte Carlo models have become the gold standard for photon transport simulations in tissue. Their flexible configuration allows for modelling of simple semi-infinite homogeneous media to complex multi-layered tissue (Prahl et al., 1989; Wang et al., 1995) with inclusions simulating blood vessels (Jones et al., 2017b) or lesions (de Jode, 2000). This offers advantages in accuracy over competing simulation and mathematical modelling techniques such as those based on diffusion theory (Flock et al., 1989) and it has been widely applied in biomedical photon propagation simulation problems (Zhu and Liu, 2013; Periyasamy and Pramanik, 2017). In spectrally-resolved imaging studies MC modelling has shown particular utility in estimating the wavelength dependence of the *DPF* (Ma et al., 2016a; Thatcher et al., 2016) and in generating inverse models to extract quantitative tissue optical properties *via* error minimisation (Saccomandi et al., 2016; Wirkert et al., 2016).

More recently, Wirkert et al. (2016) and Ayala et al. (2019) have used MC simulation to quantify the performance of a random forest estimator using varying noise and SNR conditions, and demonstrated improved accuracy data compared to least-squares regression. They were also able to optimise the computationally-expensive training step by demonstrating that the absolute error stabilised after 10^4^ training samples. Jones et al. (2017b) used a mesh-based Monte Carlo model, based on blood and submucosa optical properties, to generate a synthetic reflectance dataset. This allowed comparison of an RGB-based oxygen saturation estimation algorithm with a multispectral Beer-Lambert-based regression. The simulation results were convolved with known spectral sensitivity curves of a standard colour camera and an LCTF MSI system to generate digital datacubes, demonstrating an improvement in prediction accuracy of 10% in the RGB result when using Tikhonov regularisation.

It is essential that these MC models are capable of encapsulating the full range of real, physical, variability in tissue if accurate measures of physiological properties can be inferred. Principal component analysis provides a useful measure of assessing how well real data can be explained by the model. Wirkert et al. (2017) were able to demonstrate that 97% of the variance in their *in vivo* data lay on the simulated data's first three principal components. It was also illustrative to see that one particular organ, the gallbladder, was an exception and fell outside this, indicating a limitation in the MC simulation (possibly consideration of bile as a significant absorber). Similarly, Styles et al. (2006) showed that simulation results did not encompass the space occupied by *in vivo* data from fundus imaging experiments, but a correction in the form of an empirical scaling factor could correct the problem.

### 4.2 Phantom and *in vitro*

A common first step in validation of spectral measurement accuracy is to test the imaging system against a set of standardised targets with varying spectral properties, such as a Macbeth-type colour-checker card (Clancy et al., 2016b, 2018; Wang et al., 2018). These provide a set of calibrated colour tiles with smoothly-varying reflectance spectra. A mean spectrum for a small region-of-interest within each tile is calculated and compared to a gold standard spectrum obtained with a high-resolution spectrometer. This allows researchers to assess the performance of the SSI system across the spectral range. These targets are useful for establishing device baseline spectral accuracy and SNR, although the broad nature of the reflectance features make them unsuitable for quantifying spectral resolution or evaluating spectral unmixing. An estimate of an SSI system's ability to recover chromophore concentrations on a diverse background of optical loss mechanisms can only be achieved with more realistic tissue models.

**Fig. 5:**
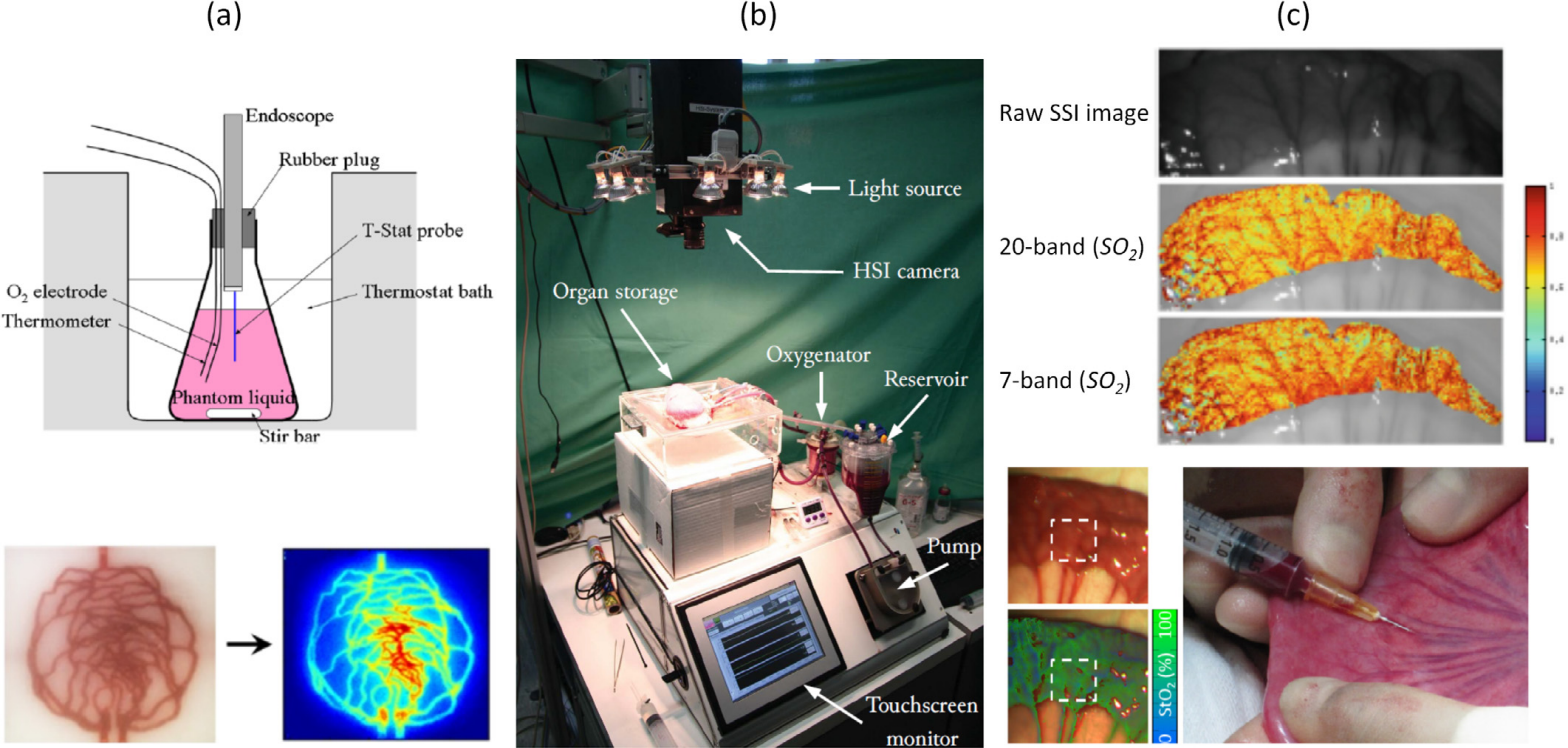
Validation. (a) Top: haemoglobin-based liquid phantoms can be temperature and oxygenation controlled and have the gold standard measurement probes placed *in situ* (top) (Saito and Yamaguchi, 2015). Adapted, with permission, from Saito and Yamaguchi, Optical imaging of haemoglobin oxygen saturation using a small number of spectral images for endoscopic application, J. Biomed. Opt. 20, 2015. Bottom: this blood can be pumped through 3D-printed flow phantoms and used to verify perfusion maps (Ghassemi et al., 2015). Adapted, with permission, from Ghassemi et al., Rapid prototyping of biomimetic vascular phantoms for hyperspectral reflectance imaging, J. Biomed. Opt. 20, 2015. (b) Machine perfusion of solid organs enables a very close simulation of *in vivo* conditions, allowing control over oxygen levels and temperature (Tetschke et al., 2016). Image reproduced with permission from the authors. (c) Top: *in vivo* SSI data can be used to validate hardware optimisation strategies such as band selection techniques, comparing them to a hyperspectral dataset (Wirkert et al., 2014). Reprinted by permission from Springer Nature Customer Service Centre GmbH: Springer Lecture Notes in Computer Science, (Endoscopic Sheffield index for unsupervised *in vivo* spectral band selection, Wirkert et al.) © (2014). Bottom: blood gas analysis can be used as a gold standard SO_2_ reference for samples withdrawn from a corresponding SSI-imaged region-of-interest. Adapted, with permission, from (Clancy et al., 2015), Optical Society of America.

*In vitro* models with tuneable optical properties provide an opportunity to test more complex functionality of the imaging system under more realistic, but still controlled, conditions (Fig. 5(a)). These so-called phantoms can, in their simplest form, be dye solutions to test spectral unmixing methods (Pichette et al., 2016) and the ability of the system to resolve the spatial location of different chromophores. More complex and physiologically-realistic optical properties can be achieved with multi-layer agar-based phantoms, with absorption and scattering properties set using India ink and intralipid, respectively (Nishidate et al., 2011, 2013). More relevant phantoms incorporate blood or haemoglobin, and include reference gas probes in tandem with temperature, pH, flow and oxygenation control (Saito and Yamaguchi, 2015; Sakota et al., 2015; Tetschke et al., 2016; Gehrung et al., 2019). Realistic models include both solid and liquid elements, with simulated vessels running through an agarose tissue (Luthman et al., 2018). The simulated vessels can further be formed into complex patterns, using rapid prototyping techniques, based on images of real vascular networks (Ghassemi et al., 2015). The phantom material's temporal stability must be considered if repeat measurements are needed, as agar and gelatine are vulnerable to decay and bacterial growth without specialist storage and treatment (Pogue and Patterson, 2006). Alternative materials such as gelwax (Maneas et al., 2018), polyvinyl chloride plastisol (Fonseca et al., 2016), silicone (de Bruin et al., 2010) and polymer gels (Cabrelli et al., 2017) are capable of offering long-term stability. The complexity of these models does not typically extend to include confounding absorbers, such as bilirubin, or complex structures, such as colonic crypts, that may affect measured spectra. Apart from flow, dynamic effects such as peristalsis or inflammatory response are also difficult to replicate.

### 4.3 Ex vivo

Spectral imaging systems are increasingly being looked to as potential tools for *optical biopsy*. That is, to obtain quantitative diagnostic information which is currently the preserve of histological analysis. Diagnosis of disease in its early stages or separation of lesions depending on their malignant potential are examples of such problems. In these cases the lesions may be small and superficial, and their appearance may be indistinguishable from surrounding normal tissue under white light illumination. Investigation of potential optical sensitivity to these pathologies, with their myriad biochemical differences, is outside the scope of synthetic models and can only be accomplished in real tissue. The initial step is to form a picture of the optical properties of each tissue type of interest. Assuming there is no *a priori* knowledge this means acquiring high resolution spectral data at as many wavelengths as possible. These data can then be analysed to determine where spectral differences may lie.

However, depending on the tissue in question, the ability to obtain *in vivo* data may be limited due to challenging experimental conditions. In these cases exploratory data is usually acquired from tissue after it has been resected. The advantages of this are that data collection has minimal impact on the surgical workflow, imaging can be performed in a controlled, motion-free, environment, and the sample will subsequently proceed to pathology for ground truth histological confirmation. This approach has been used to study the spectral characteristics of cancers in head & neck (Akbari et al., 2012), colon (Leavesley et al., 2016) and pancreas (Kiris et al., 2015). The major limitation is that the blood supply is immediately cut off once the tissue is excised. Given the influence of haemoglobin on tissue reflectance, the effect on the optical properties may be significant and unpredictable. Disease-specific signatures based on oxygenation become less distinct. A complex series of events characterise tissue degradation and drift in tissue optical properties in the first few hours post excision, including osmosis, shrinkage and ischaemia-induced scattering changes, depending on how the sample is stored (Hsiung et al., 2005).

Machine perfusion techniques, developed to preserve harvested organs for transplantation, may offer a potential solution to some of the challenges associated with validation of SSI in physiologically realistic scenarios (Fig. 5(b)). These machines pump blood through the organ's vessels while maintaining normothermic conditions and can allow oxygenation control (Tetschke et al., 2016). Careful preparation of the organ in question is required, using similar protocols to transplantation retrieval, to ensure minimal damage to the tissue.

### 4.4 In vivo

Intraoperative validation remains a challenging task due to the limited control over physiological variables and multiple sources of measurement noise from both biological signals and motion-induced artefacts. Nevertheless this is an essential testbed in the developmental cycle of a surgical imaging system, providing insight into its future clinical utility. One approach to mitigating the influence of the aforementioned complications is to temporally measure relative changes in response to a stimulus, such as a vessel occlusion, and monitor the instrument's response. This has been reported in imaging experiments, following mechanical occlusion, on the skin (Nishidate et al., 2013), bowel (Clancy et al., 2015) and kidney (Best et al., 2011). Similar induction of hypoxia has also been achieved in rodents by restricting the fraction of inspired oxygen (Akter et al., 2017; Nishidate et al., 2018). Estimation of in-band noise and camera signal-to-noise ratio were made by Wirkert et al. (2016) during vascular occlusion of a pig bowel segment and correlated to *in silico* experiments.

Absolute quantification of tissue constituents *in vivo* is more difficult due to the lack of gold standard measurement modalities that are directly comparable to optical imaging results. Perhaps the most recognisable clinical oxygen saturation device, the pulse oximeter, provides measurements of systemic arterial, rather than local tissue, oxygenation. Machines for biochemical analysis of blood, such as the co-oximeter, are another frequently used clinical tool, and can provide detailed biochemical information. This includes gas partial pressures, haemoglobin concentrations and lactate levels, which is a surrogate marker of oxygenation. Imaging results have been validated using blood gas analysis from locally-drawn samples (Clancy et al., 2015) and, in an SFDI study, calibrated optical oxygenation probes (Gioux et al., 2011). Interpretation of these types of single-point validation measurements is inevitably complicated by the fact that each technique probes a different volume of tissue, leading to some deviation in absolute values, although temporal trends agree. The problem is especially acute for blood gas analysis, where even microlitre-scale samples must be drawn from a comparatively large vessel that is not necessarily representative of the mixture of arterial and venous microvessels running through the organ-of-interest.

Models for delineating tumour boundaries have been evaluated in terms of accuracy (Panasyuk et al., 2007), and true and false positive rates when compared to histological gold standard and manually-segmented images (Panasyuk et al., 2007; Han et al., 2016). *In vivo* classification accuracy has also been assessed, using leave-one-out cross-validation (LOOCV), for an SVM-based algorithm to identify atherosclerotic plaques (Chihara et al., 2016) and deep neural network methods to predict tissue reflectance spectra (Lin et al., 2018b). While diagnostic accuracy in a single lesion can be evaluated in this way, there is still a question over the accuracy of the margin delineation. This is due to the small sample size of the gold standard, which ultimately is derived from a biopsy section a few millimetres in diameter.

Despite the difficulties associated with *in vivo* validation of optical properties, intraoperative MSI and HSI datasets are well-suited to testing the performance of computational RGB-to-SSI approaches. In these cases the spectral datacubes themselves can be treated as the ground truth (Fig. 5(c)). Jones et al. (2017a) derived a test set of RGB images from an intraoperative MSI dataset using the spectral sensitivity curves of a standard colour camera. The proposed computational method predicted SO_2_ with mean error less than 10%, compared to the MSI result. A similar approach was used by Lin et al. (2018b) to test the accuracy of a deep neural network spectral reconstruction algorithm, showing that mean relative errors as low as 0.63% between predicted and reference spectra could be achieved. Nishidate et al. (2013) incorporated a beam-splitter in the set-up to obtain reflectance spectra from the surface of the skin in parallel with RGB measurements, enabling them to demonstrate qualitative similarity between the spectra with an average relative SO_2_ estimation error of 54.5%.

There are some well-known resources for established medical imaging modalities such as CT/MRI where researchers may access annotated image data. Examples include The Cancer Imaging Archive (TCIA; https://www.cancerimagingarchive.net/) (Clark et al., 2013), open-CAS (http://opencas.webarchiv.kit.edu), the EndoVIS challenge (https://endovis.grand-challenge.org) and DeepLesion (https://nihcc.app.box.com/v/DeepLesion) (Yan et al., 2018). The largest of these databases is still 3–4 orders of magnitude smaller than natural image datasets such as ImageNET (http://www.image-net.org). SSI is lagging much further behind still and there are very few publicly-available datasets. As an initial step researchers in the field should make their data available on institutional servers, following the example of groups working in endoscopy (Yoon et al., 2019a, 2019b), neuroimaging (Fabelo et al., 2019b, 2019a), ophthalmology (A. Gebejes et al., 2016, 2016) and histology (Awan et al., 2018b, 2018a). When published, these data should be accompanied by a readme file containing a brief description of the study and associated protocol, links to related publications and a clear statement on any usage restrictions. The key aim is to enable an external researcher to reproduce the results of the original team using the information supplied.

While a DICOM-equivalent standard for SSI may still be some way off, metadata, either embedded or stored as a separate file, should be included with each acquisition. A suggested format is to include Patient/Subject ID (anonymised), timestamp, spectral channel information (e.g., central wavelength, band FWHM, pixel-to-wavelength calibration data [for push/whiskbroom systems]), camera/detector settings (exposure time, gain, bit depth), reference reflectance spectrum and background intensity measurements. For SSI systems where the spectral characteristic of a particular channel is not easily described by a Gaussian shape, the filter/illumination spectrum data or a link to manufacturer specifications should be provided. A second layer of related information could include image annotations, either in the form of regions-of-interest with defined co-ordinates or binary masks to delineate separate organs, tissues or lesions. Labels linked to these spatial data would identify the feature in question and/or the results of histological evaluation. Beyond this, future large-scale curated imaging databases will require national or international initiatives with dedicated funding and resources to maintain submissions and ensure quality control. Following the TCIA template this would allow for rigorous validation, version control and accommodation of corroborating data such as histology slides, clinical information or other medical imaging modalities.

## 5 Discussion

Spectral imaging has become recognised as a valuable method of obtaining functional and structural information on tissue non-invasively. It has gained widespread uptake in clinical research, with the list of applications now spanning dermatology, gastroenterology, gynaecology, otolaryngology, cardiology, haematology, neurology, ophthalmology, bronchoscopy, nephrology and hepatology (Table 1). Strong contrast due to haemoglobin has enabled imaging of blood volume and oxygenation, both important variables in the assessment of tissue perfusion and its ability to recover from injury. Physiological processes, such as tumour-associated angiogenesis or the proliferation of dysplastic cells, can be detected through their impact on the absorption and scattering components of the tissue's reflectance spectrum. Selection of wavelengths that maximise the spectral difference between organs or pathologies can be used to optimise SSI device design, and solve image segmentation and classification problems. Statistical and computational tools have allowed this process to be optimised, increasing robustness and resolving differences not appreciable under standard white-light illumination, for example, differentiating the thyroid and parathyroid (Barberio et al., 2018). Its usefulness as a collaborative tool has also been demonstrated, providing valuable context for other imaging modalities and showing potential in assisting guidance and clinical decision-making.

Spectral imaging has reached something of a crossroads in the surgical field. Although the principles of the technique have long been established the number of attempted clinical studies has recently increased rapidly. Fuelled by growing industrial and remote-sensing demand, readily-available computational power and newly-developed software techniques, truly useful SSI devices may soon be ready to make the leap into clinical practice.

The main factors for translation of a new imaging modality include three interconnected problems: clinical validation, usability and ease-of-interpretation. While SSI has certainly demonstrated utility across a wide range of specialties and is clearly sensitive to a variety to relevant biological variables (Table 1), it has yet to address the crucial question of whether or not it can improve outcomes for the patient. This is the difficult next step for the field as it requires collection of a large amount of data in carefully-designed clinical studies. Despite the number of publications on the subject very few *in vivo* studies have been conducted (Shapey et al., 2019), most have small sample sizes and are not correlated with surgical outcomes. For example, while many studies show sensitivity to tissue perfusion and oxygenation none can conclusively estimate the range of SSI values that represents a ‘healthy’ blood supply. Similarly, there is undoubted applicability in tissue classification and disease detection, but no study that proves that SSI can have a significant impact on tumour recurrence rates. Clinical efficacy studies are needed to answer these questions.

Collection of data to answer clinical efficacy questions requires studies on a larger scale than are currently being attempted. This places demands on hardware performance and robustness, and connects to the second of the aforementioned problems, which includes device resolution, speed and mechanical properties. That is, a new technology should not compromise the surgeon's vision of the tissue, or their ability to navigate the internal anatomy safely, and should have equivalent handling characteristics, i.e., weight, size, rigidity, to standard tools. Acquisition and processing speed are important, to avoid delays to the normal delivery of care to the patient. This problem may be on the cusp of being solved, as the gap between high resolution and high-speed systems is becoming less distinct. Table 2 shows that commercially-available snapshot sensors can now capture MSI datacubes comprising 6–25 spectral bands at up to 90 fps. There are also cameras, such as IMEC's *Snapscan*, that sacrifice some speed to achieve hyperspectral detection at megapixel spatial resolution. These devices are monolithic in design, with single sensor snapshot cameras in particular offering a light weight option. This means that they can be integrated in existing surgical optical imaging devices, such as laparoscopes and operating microscopes, in an equivalent manner to standard colour cameras.

The third problem emphasises that the processing results should be displayed in a concise and efficient manner. i.e., SSI should not increase the cognitive burden on the surgeon. The solution to this lies in efficient and accurate computation of physiological variables, delineation of diseased tissue and intuitive display of the information to the clinician. The question of the optimal way to relay this information to the surgeon remains open. Existing scopes with enhanced imaging facilities typically allowing the user to toggle between different modes or to choose an overlay. Augmented reality systems (Bernhardt et al., 2017) may be one platform to deliver this information, allowing the surgeon to seamlessly switch to SSI or another imaging modality overlay during a procedure.

The range of acquisition systems summarised in Table 1 and the nuances of their individual characteristics illustrates the full range of complexity open to a researcher in acquiring data for a specific need. Unfortunately, it has resulted in a disparate dataset, lacking standardisation, that has hindered development of efficient computational algorithms and independent validation and benchmarking. The robustness of processing algorithms based on learning will depend on the reliability of the training data available. This is especially important as the reflectance spectral signatures of tissue constituents are broad, and the ability of SSI devices to identify ‘fingerprints’ of specific compounds is limited. A well-documented SSI dataset is essential in understanding the applicability and limits of any newly-developed algorithm. Furthermore, built-in algorithmic awareness of variance and uncertainty in a particular model's performance will help to ease safety concerns, by restricting diagnostic decisions to those based on ‘high confidence’ measurements.

Computational spectral estimation methods using RGB data are an exciting proposition as they have the potential to bridge the gap between high resolution and high-speed devices, and introduce the possibility of software-enabled SSI from conventional cameras. Accuracy remains a challenge due to the inherent low spectral resolution of RGB which, at ~100 nm, washes out fine spectral features that distinguish, for example, oxygenated and deoxygenated haemoglobin. Model and AI-based approaches will need to be tested across a wider variety of image data to avoid overfitting to a narrow range of physiological properties. An SSI imaging database, from which RGB images can also be synthesised, of multiple test subjects, organs and pathologies matched with histology is needed. This is a field that may become more important as rigid endoscopes begin to transition from rod lenses to chip-on-tip. Some of the major endoscope manufacturers already have products in this area, such as Karl Storz (TIPCAM, C-MAC VS) and Olympus (Endoflex 3D), and more can be expected as sensor miniaturisation continues.

Idealised databases are challenging to create, requiring monetary and resource investment in a multi-centre collaboration. The time taken to design, set up and conduct the required measurements is significant. However, previous medical imaging work using transfer learning (Litjens et al., 2017) has shown that this task can be simplified by using networks trained for other tasks or on different samples (Lin et al., 2017). This field includes methods that can be trained on synthetic data and, with minimal retraining, be reconfigured to work on real data or a completely different scenario. Thus SSI results could be generalised more easily and each new clinical application would not require a separate large-scale study to obtain sufficient annotated training data (Wirkert et al., 2017).

SSI could also take some inspiration from fluorescence image-guided surgery (FIGS), which has recently enjoyed an explosion of interest, particularly using near-infrared light which does not interfere with the standard colour imaging. Despite its relative complexity, which requires exogenous agents and use of a specialised camera system, there are now several general systems available for use in open, MIS and robotic-assisted MIS. These clinically-approved devices are being used in several studies to explore the full utility of the technique and determine its efficacy in improving surgical procedures. While clinical spectral imaging systems do exist, aimed principally at dermatology, a MIS-compatible camera is not currently available. This will be needed to instigate long-term clinical studies, especially for applications where the problem is significant but the incidence is low.

Health economics will play a significant role in whether or not SSI devices are widely adopted in practice. Ultimately the new technology will have to demonstrate that its cost can be recovered. Miniaturised spectral imaging sensors, although increasingly available, are still approximately five orders of magnitude more expensive that standard colour sensors. Given the scale of expense associated with some of the clinical problems mentioned here, such as management of patients following anastomotic leaks, transplant failure, or unnecessary removal of hyperplastic lesions, a convincing financial case for spectral imaging devices might still be formed despite the high initial outlay. Furthermore, SSI methods can point to a major advantage over current surgical imaging competitors, fluorescence-guidance, in that running costs related to purchase of dyes do not apply. Thus, while SSI devices are unlikely to become similarly priced to RGB hardware due to the economy of scale of manufacturing, they may at least become a realistic competitor to FIGS. This remains an open question, and one that requires appropriately-structured clinical trials to yield an answer.

The above challenges are echoed by recent attempts to formalise the translational route for new optical technologies (Waterhouse et al., 2019). This notes, in addition to the points already discussed here, that there is a general problem with a lack of standardisation in optical techniques, including metrics of exposure safety limits.

## 6 Conclusions

Interest in spectral imaging for clinical applications continues to grow along with the variety and performance of the technology. Potential uses have been found in guidance, viability monitoring and disease-detection, exploiting endogenous contrast in the visible and near-infrared wavelength range. Increased commercial interest has seen the development of lightweight, snapshot devices equivalent in size to standard cameras that can now be mounted on endoscopes or operating microscopes, while high-resolution hyperspectral devices can be mounted on articulated arms. Advances in computational modelling, including the use of statistical techniques and deep learning, has augmented the hardware to increase speed and accuracy. Nevertheless, some significant challenges remain.

While SSI has seen increased use in research, most studies are small or at proof-of-concept stage and there is still no clinical application where the technique is used routinely. Clinical trials are required to establish correlations between SSI signals and surgical outcomes. Demonstrating the potential health and economic impact of the technology will not be possible without this.

To reach this milestone some hardware refinements are still needed to provide seamless switching between real-time colour visualisation and spectral acquisition, avoiding the need for expert set-up and maximising clinical uptake. The system must provide equivalent white light imaging performance to currently-used medical cameras to avoid disruption of the clinical workflow and patient care. Current surgical imaging configurations, particularly in MIS, vary widely, are highly application-specific and commercial systems are not yet optimised for this task.

Creation of standardised databases of SSI sequences, with accompanying acquisition and calibration metadata, will be essential to allow benchmarking of processing algorithms, development of computational techniques and independent validation of spectral measurements. This will become increasingly important with the growing trend toward data-driven processing. This will help to establish validation of the measurement technique *in vivo* in addition to any *in silico* and *ex vivo* experiments. Computational spectral-from-RGB estimation imaging techniques, which are still in their infancy, would benefit from these databases, allowing researchers to increase their robustness and applicability.

These challenges must be met in collaboration with both industrial and clinical partners. A consistent and robust hardware set-up will allow reliable data acquisition across multiple hospital departments and sites, maximising the impact of any study that is conducted.

## CRediT authorship contribution statement

**Neil T. Clancy:** Writing - original draft, Writing - review & editing, Methodology, Data curation. **Geoffrey Jones:** Writing - original draft, Writing - review & editing. **Lena Maier-Hein:** Writing - review & editing. **Daniel S. Elson:** Writing - review & editing. **Danail Stoyanov:** Writing - review & editing.

## Declaration of Competing Interest

None.
